# Seizure-Induced Potentiation of AMPA Receptor-Mediated Synaptic Transmission in the Entorhinal Cortex

**DOI:** 10.3389/fncel.2018.00486

**Published:** 2018-12-11

**Authors:** Dmitry V. Amakhin, Elena B. Soboleva, Julia L. Ergina, Sergey L. Malkin, Anton V. Chizhov, Aleksey V. Zaitsev

**Affiliations:** ^1^Laboratory of Molecular Mechanisms of Neural Interactions, Sechenov Institute of Evolutionary Physiology and Biochemistry of the Russian Academy of Sciences, Saint Petersburg, Russia; ^2^Ioffe Institute, Russian Academy of Sciences, Saint Petersburg, Russia; ^3^Institute of Experimental Medicine, Almazov National Medical Research Centre, Saint Petersburg, Russia

**Keywords:** temporal lobe epilepsy, calcium-permeable AMPA receptor, epileptiform activity, entorhinal cortex, seizure-like event, synaptic plasticity

## Abstract

Excessive excitation is considered one of the key mechanisms underlying epileptic seizures. We investigated changes in the evoked postsynaptic responses of medial entorhinal cortex (ERC) pyramidal neurons by seizure-like events (SLEs), using the modified 4-aminopyridine (4-AP) model of epileptiform activity. Rat brain slices were perfused with pro-epileptic solution contained 4-AP and elevated potassium and reduced magnesium concentration. We demonstrated that 15-min robust epileptiform activity in slices leads to an increase in the amplitude of α-amino-3-hydroxy-5-methyl-4-isoxazolepropionic acid receptor (AMPAR)-mediated component of the evoked response, as well as an increase in the polysynaptic γ-aminobutyric acid (GABA) and *N*-methyl-D-aspartate (NMDA) receptor-mediated components. The increase in AMPA-mediated postsynaptic conductance depends on NMDA receptor activation. It persists for at least 15 min after the cessation of SLEs and is partly attributed to the inclusion of calcium-permeable AMPA receptors in the postsynaptic membrane. The mathematical modeling of the evoked responses using the conductance-based refractory density (CBRD) approach indicated that such augmentation of the AMPA receptor function and depolarization by GABA receptors results in prolonged firing that explains the increase in polysynaptic components, which contribute to overall network excitability. Taken together, our data suggest that AMPA receptor enhancement could be a critical determinant of sustained status epilepticus (SE).

## Introduction

Status epilepticus (SE) is a common emergency condition with a considerable mortality rate (Betjemann and Lowenstein, 2015) that can result from the activation of currently unknown mechanisms that sustain seizures (Joshi and Kapur, 2018). Some features of epileptic seizures and SE can be reproduced using *in vitro* models of epileptiform activity, such as the 4-aminopyridine (4-AP) model in rat brain slices that contain both hippocampus and entorhinal cortex (ERC; Avoli and Jefferys, 2016). The implementation of this model allows for inducing multiple seizure-like events (SLEs) in rat brain slices, which makes it a convenient tool for studying the mechanisms of abnormal neuronal synchronization (Avoli et al., 2002). It is generally accepted that the balance between excitation and inhibition shifts towards excitation during epileptic seizures, and most studies point towards a decrease in the efficiency of γ-aminobutyric acid (GABA)a-receptor (GABAaR) mediated inhibition as a primary cause of this imbalance (mainly due to intracellular chloride accumulation; Barmashenko et al., 2011; Glykys et al., 2014; Alfonsa et al., 2015; Raimondo et al., 2015); however, an increasing amount of evidence indicates that the potentiation of α-amino-3-hydroxy-5-methyl-4-isoxazolepropionic acid receptor (AMPAR)-mediated synaptic transmission to hippocampal neurons can develop due to seizures (Abegg et al., 2004; Debanne et al., 2006; Joshi et al., 2017). Currently, the role it plays in maintaining the SE and the long-term consequences of SE-induced changes in the brain circuitry are not fully understood.

During epileptiform activity in brain slices, high numbers of neurons fire synchronously and receive a roughly identical synaptic input (Amakhin et al., 2016). The sophisticated interplay of excitatory and inhibitory input signals results in specific firing patterns, which manifest as interictal and ictal discharges under *in vitro* conditions (Fujiwara-Tsukamoto et al., 2004; Ziburkus et al., 2006; Žiburkus et al., 2013). In previous studies, the seizure-induced changes in AMPAR-mediated transmission were investigated either after a period of epileptiform activity evoked by the blockade of GABAaRs (Abegg et al., 2004; Debanne et al., 2006) or after a time interval after SE, which was required for animal sacrifice and the preparation of slices (Rajasekaran et al., 2012; Joshi et al., 2017). In the first case, the GABAaR-mediated input to the pyramidal neurons does not occur, which is not the case for seizures in patients. In the latter case, the potential changes in synaptic transmission properties can be underestimated as the potentiation of AMPARs dissipates over time after the termination of SEs. In some studies on the plastic changes of AMPAR-mediated transmission, the induced epileptiform activity in a model object or epileptic seizures in animals lasted much longer than that it could be supposed to occur in patients (Abegg et al., 2004), which can lead to much stronger plastic changes in the excitatory synapses than is possible in clinics.

The ERC is highly involved in the generation of seizures in patients with temporal lobe epilepsy (Vismer et al., 2015), the most common form of epilepsy in humans. In this study, using a 4-AP model of epilepsy in rat brain slices, epileptiform activity induced alterations of synaptic transmission in ERC pyramidal neurons were investigated. The simultaneous activation of both GABA- and glutamate-receptor mediated responses by an extracellular stimulus allowed for investigating the changes in the interplay of these synaptic input signals. The implementation of the synaptic conductance estimation algorithm allowed for investigating these changes without interrupting or pharmacologically altering ongoing epileptiform activity induced by the interplay of both GABAergic and glutamatergic neurons. In a comparison between ERC and prefrontal cortex (PFC), the strengthening of AMPAR-mediated synaptic responses in ERC pyramidal neurons occurred, which is *N*-methyl-D-aspartate receptor (NMDAR)-dependent and persists for some time after the cessation of induced seizures. Using a conductance-based refractory density (CBRD) approach to model neuronal populations, it was demonstrated that the enhancement of the AMPAR function in pyramidal neurons might be a key factor that determines increased network excitability during epileptiform activity.

## Materials and Methods

### Animals

The experiments were carried out on 3-week-old Wistar rats of both sexes (*n* = 65). All animal procedures followed the guidelines of the European Community Council Directive 86/609/EEC and were approved by the Animal Care and Use Committee of the Sechenov Institute of Evolutionary Physiology and Biochemistry of the Russian Academy of Sciences.

### Slice Preparation

Rats were sacrificed by decapitation, and their brains were removed rapidly. The brain slice preparation was described previously in detail (Amakhin et al., 2017). A vibrating microtome (Microm HM 650 V; Microm; Germany) was used to cut either horizontal 300-μm-thick slices that contained the ERC and hippocampus or coronal 300-μm-thick slices that contained the PFC. Artificial cerebrospinal fluid (ACSF) with the following composition was used for all steps (in mM): 126 NaCl, 24 NaHCO_3_, 2.5 KCl, 2 CaCl_2_, 1.25 NaH_2_PO_4_, 1 MgSO_4_, and 10 dextrose. The ACSF was aerated with a gas mixture of 95% O_2_ and 5% CO_2_.

### *In vitro* Model of Epileptiform Activity

Epileptiform activity in ERC-containing slices was induced using a pro-epileptic solution (Amakhin et al., 2016; Chizhov et al., 2017, 2018) containing the following (in mM): 120 NaCl, 8.5 KCl, 1.25 NaH_2_PO_4_, 0.25 MgSO_4_, 2 CaCl_2_, 24 NaHCO_3_, 10 dextrose, and 0.05 4-AP. The flow rate in the perfusion chamber was 5–6 ml/min. In this study, only the slices that generated at least three SLE were utilized for further analysis. In the case of evoked postsynaptic currents (ePSCs) recordings during epileptiform activity, the stimuli were applied between spontaneous discharges (at least 700 ms after the last event).

Miniature excitatory postsynaptic currents (mEPSCs) were recorded before and after the induction of epileptiform activity in ERC slices. Epileptiform activity was induced by the hour-long incubation of the slices in the pro-epileptic solution at 30°C. The slices were then transferred to the ACSF-perfused recording chamber for mEPSC registration.

In some experiments, a modified model of *in vitro* epileptiform activity in which only excitatory synaptic conductances could be activated was used. In this case, the same pro-epileptic solution was used with the addition of either bicuculline or bicuculline in combination with NMDAR antagonists (MK-801, AP-5, CNS-1102). In both cases, a similar pattern of epileptiform activity was induced (see the “Results” section).

### Patch-Clamp Recordings in Rat Brain Slices

The recordings were performed at 30°C. Pyramidal neurons in the deep layers of the medial ERC or layer 2/3 of the PFC were visualized using a Zeiss Axioskop 2 microscope (Zeiss, Germany) equipped with differential interference contrast optics and a video camera (PointGrey Grasshopper 3 GS3-U3-23S6M-C, FLIR Integrated Imaging Solutions Inc., USA). Patch electrodes (2–4 MΩ) were pulled from borosilicate glass capillaries with filaments (Sutter Instrument, One Digital Drive Novato, CA, USA) on a P-1000 Micropipette Puller (Sutter Instrument, One Digital Drive Novato, CA, USA). A cesium-methanesulfonate-based solution was used for the whole-cell voltage-clamp recordings of the ePSCs; composition, in mM: 127 cesium methanesulfonate (CsMeSO_4_), 10 NaCl, 5 ethylene glycol-bis (β-aminoethyl ether)-*N*,*N*,*N*′,*N*′-tetraacetic acid (EGTA), 10 4-(2-hydroxyethyl)-1-piperazineethanesulfonic acid (HEPES), 6 QX314, 4 ATP-Mg, and 0.3 GTP. The pH level was adjusted to 7.25 with CsOH. In some experiments for which the intracellular blockade of NMDARs was required, MK-801 (3 mM, Alomone Labs, Israel) was added to the pipette solution. For the cell-attached voltage-clamp recordings, a sodium-chloride-based solution was used. The composition in mM was as follows: 138.5 NaCl, 8.5 KCl, 10 HEPES, and 5 EGTA. The pH level was adjusted to 7.25 with NaOH. The cell-attached voltage-clamp recording of neuron firing activity was performed as described previously (Perkins, 2006). For the recordings of the mEPSCs, a potassium-gluconate-based solution was used with a composition in mM as follows: 114 K-gluconate, 6 KCl, 0.2 EGTA, 10 HEPES, 4 ATP-Mg, and 0.3 GTP. The pH level was adjusted to 7.25 with KOH.

The recordings were performed using a Model 2400 (AM-Systems, Sequim, WA, USA) patch-clamp amplifier, an NI USB-6343 A/D converter (National Instruments, Austin, TX, USA) and WinWCP 5 software (SIPBS, UK). The data were filtered at 10 kHz and sampled at 20 kHz. For whole-cell recordings in all cells included in the sample, access resistance was less than 15 MΩ, and it remained stable (≤20% increase) across the experiments. The liquid junction potential for the CsMeS0_4_-based pipette solution was estimated as described previously (Neher, 1992), and the holding potential was compensated for offline for voltage-clamp recordings by subtracting 7 mV. No compensation was performed for the potassium gluconate-based solution, as the recordings were performed on the same constant level of holding voltage.

The synaptic responses were evoked extracellularly. The stimulating bipolar electrode (WPI Inc., Blacksburg, VA, USA) was placed in the same layer of the cortex as the recorded neuron at a distance of 100–200 μm. ePSCs were recorded at various holding voltages from +43 mV to −97 mV with a step of −10 mV. AMPAR-mediated evoked excitatory postsynaptic currents (eEPSCs) were recorded at −90 mV in the presence of either only bicuculline (20 μM, Tocris Bioscience, UK) or bicuculline in combination with NMDAR-antagonists MK-801 (10 μM in extracellular solution and 3 mM in pipette solution, Alomone Labs), AP-5 (50 μM, Tocris Bioscience), and CNS-1102 (3 μM, Alomone Labs). In some pyramidal cells, the rectification index, calculated as AMPAR-mediated eEPSC amplitude ratio at +50 mV and −50 mV holding voltages, was estimated. For these experiments, spermine (100 μM) was added to a CsMeSO_4_-based pipette solution to prevent the loss of rectification of CP-AMPARs during the recordings (Kamboj et al., 1995). For the comparison between the recordings at different holding voltages, the AMPAR-mediated evoked currents were recalculated into the units of conductance (nS) using the following equation:

(1) $$g_{\text{AMPA}}=\frac{I_{\text{eEPSC}}}{\left(U-V_{\text{AMPA}}\right)}$$

where *I*_eEPSC_ is the amplitude of the eEPSC (in pA), *U* is the holding voltage (in mV), and *V*_AMPA_ is the reversal potential of the AMPAR-mediated current (≈+3 mV for CsMeSO_4_ based pipette solution).

The input-output (I/O) curve—the dependence of eEPSC amplitude on stimulation strength—was determined by increasing the current intensity from 0.025 mA to 2 mA via an A365 stimulus isolator (WPI Inc., Blacksburg, VA, USA). The obtained curves were fitted with a sigmoid Gompertz function:

(2) $$g=g_{\max}e^{-e^{-k(I-X_{c})}}$$

where *g*_max_ is an asymptote of maximum evoked conductance (in nS); *e* is Euler’s Number (≈2.71828‥.); *k* is a positive number that determines the slope of the curve; *X*_c_ is a value of the current (in mA) at which the maximum slope of the curve is observed (inflection current); and the maximum slope of the curve (in nS/mA) was calculated as *g*_max_^k/e^.

The recordings of mEPSCs were performed at −80 mV in the presence of TTX (0.5 μM, Alomone Labs). NMDA and GABAa Rs were blocked with D-AP5 (50 μM, Tocris Bioscience) and bicuculline (10 μM, Tocris Bioscience) respectively. The mEPSCs were detected offline using Clampfit 10.0 software (Molecular Devices Corporation, San Jose, CA, USA), and their properties were analyzed using open-source SciPy and NumPy libraries^1^ for the Python programming language.

To obtain the average cumulative distributions of mEPSC amplitudes, the amplitudes were normalized by their mean for each cell, then the individual distributions were calculated, and the resulting cumulative probability values were averaged for each amplitude range. The two-sample Kolmogorov-Smirnov test for differences in the resulting average distributions was performed.

### Estimation of AMPAR-, GABAaR-, and NMDAR-Mediated Conductances

To assess the relative impacts of AMPA, NMDA, and GABAa Rs during the evoked responses in a neuron, the mathematical approach described in the authors’ previous studies was implemented (Amakhin et al., 2016, 2017; Chizhov and Amakhin, 2018).

For the implementation of this method, the approximations of the I-V relationships for NMDAR-, AMPAR-, and GABAaR-mediated synaptic currents are required, which were performed in the author’s previous study (Amakhin et al., 2016).

The I-V relationships of the GABAaR-mediated currents were described by the Goldman-Hodgkin-Katz current equation (Barker and Harrison, 1988; Staley and Proctor, 1999; Jedlicka et al., 2011):

(3) $$f_{\text{GABA}}(U)=\frac{{\left(zF\right)}^{2}}{RT}PS\;U\frac{[C_{\text{out}}]\left(\exp\left(\frac{-zF}{RT}(U-V_{\text{GABA}})\right)-1\right)}{\exp\left(\frac{zFU}{RT}\right)-1}$$

where *z* is the valence (−1 for chloride); F, the Faraday constant (96485 C/Mol); R, the gas constant (8.314 J·K^−1^·mol^−1^); *T*, the temperature (303 K); *C*_out_, the extracellular chloride concentration (135 mol·m^−3^); *V*_GABA_, the reversal potential (−63.8 mV for the control extracellular solution and −51.1 for the pro-epileptic solution); and *PS* (m^3^·s^−1^), the product of membrane permeability for chloride (m/s) and membrane surface area (m^2^). The value of PS was selected so that the slope of the curve was equal to 1 nS at 0 mV.

The voltage dependence of NMDA currents was approximated by the Boltzmann function (Jahr and Stevens, 1990):

(4) $$f_{\text{NMDA}}(U)=\frac{1}{1+\exp\left(\frac{V_{12}-U}{k}\right)}(U-V_{\text{NMDA}})$$

where *V*_12_ and *k* determine the voltage dependence of the Mg^2+^ block of NMDARs (*k* = 15.63 and 15.58 for the control and pro epileptic solutions, respectively; *V*_12_ = −8.65 mV and −47.77 for the control and pro epileptic solutions, respectively); *V*_NMDA_ is the reversal potential of the NMDAR-mediated current (+6.7 mV and +6.8 mV for the control and pro epileptic extracellular solutions, respectively).

The I-V relationship of the AMPAR-mediated current was considered to be linear and to have the following form:

(5) $$f_{\text{AMPA}}(U)=(U-V_{\text{AMPA}})$$

where *V*_AMPA_ is the reversal potential of AMPAR-mediated current (+2.5 mV and +3.5 mV for the control and pro epileptic solutions, respectively).

**Figure 1:**
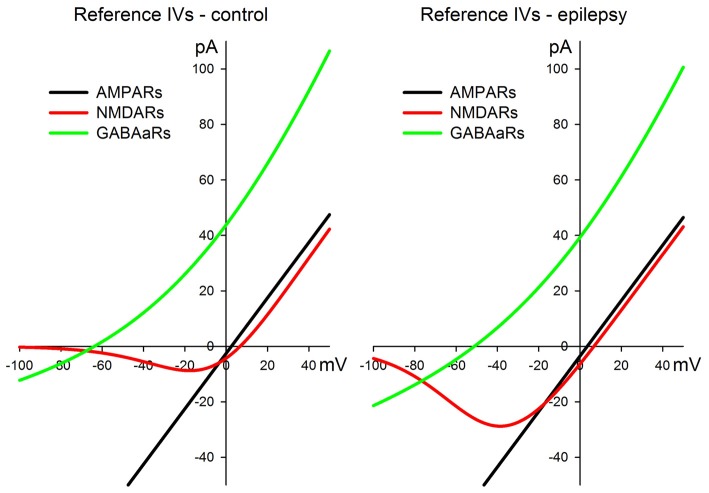
I-V relationships for the α-amino-3-hydroxy-5-methyl-4-isoxazolepropionic acid receptor (AMPAR)-, *N*-methyl-D-aspartate receptor (NMDAR)-, and γ-aminobutyric acid (GABA)a-receptor (GABAaR)-mediated currents were utilized for the estimation of synaptic conductances during the evoked responses. The left panel represents a set of curves obtained in the control artificial cerebrospinal fluid (ACSF). The right panel represents a set of curves obtained in the pro-epileptic perfusing solution. See main text for the equations and parameter values.

All the approximations for AMPARs, NMDARs, and GABAaRs are plotted in Figure 1 both for the control and pro-epileptic extracellular solutions.

Next, to estimate the synaptic conductances, we used the eEPSCs recorded at various holding potentials from +43 mV to −97 mV with a step of −10 mV. The I-V curves were obtained every 1,198 μs. AMPAR-, NMDAR-, and GABAaR-mediated conductances (*g*_AMPA_, *g*_NMDA_ and *g*_GABA_, respectively) were estimated by fitting each I-V curve obtained from the set of responses with a three-parameter function of the total current (*I*_total_):

(6) $$I_{\text{total}}(U;g_{\text{AMPA}},g_{\text{GABA}},g_{\text{NMDA}})=g_{\text{GABA}}f_{\text{GABA}}(U)+g_{\text{NMDA}}f_{\text{NMDA}}(U)+g_{\text{AMPA}}f_{\text{AMPA}}(U)$$

The least-square method was utilized to determine a reasonable estimate of conductances. Conductances were allowed to vary in a positive range only. Before fitting an offline series resistance, the compensation procedure was implemented for each obtained I-V curve.

In some cases, the area under the obtained plots of synaptic conductances was utilized to characterize the kinetics of the corresponding component of the response. The area (*A*, in nS·ms) was calculated as:

(7) $$A=1.198\cdot \sum_{t=0}^{t_{\max}}g(t)$$

where *g(t)* is the corresponding conductance of AMPARs, NMDARs or GABAaRs; *t*_max_ is 250 ms for AMPARs and 1,800 ms for NMDARs and GABAaRs.

### Offline Series Resistance Compensation Procedure

In the voltage-clamp mode, the series resistance introduces a mismatch between the holding voltage and the voltage on the membrane (Sakmann and Neher, 1995). This mismatch is proportional to the values of the holding current (*I*_total_) and the value of the series resistance (*R*_series_):

(8) $$\text{Δ}U=I_{\text{total}}\cdot R_{\text{series}}$$

Before the recordings of eEPCs, the series resistance was estimated from the response to a voltage step (from −27 mV to +43 mV). The holding voltage of each obtained IV curve was corrected by subtracting the value of the series resistance error Δ*U*, which was estimated using Eq. (7).

### Modeling: A Population-Type Model for Coupled Populations of Excitatory and Inhibitory Neurons

The model of synaptically interacting neuronal populations was based on the authors’ previous studies of interictal discharges in the ERC (Chizhov et al., 2017) and orientational selectivity and evoked responses in the visual cortex (Chizhov, 2014). Excitatory and inhibitory neuronal populations were considered. The mathematical description of each single population is based on the probability density approach, namely, the CBRD approach (Chizhov and Graham, 2007, 2008). The CBRD approach has been chosen to provide both a biophysically detailed description of a single neuronal population in terms of ionic channel conductances for two-compartment neurons and a good precision for the non-equilibrium regimes of ensemble activity. The approach considers a population of an infinite number of Hodgkin-Huxley-like neurons receiving a common input and an individual noise. In an arbitrary case of transient or steady-state stimulation, the firing rate of such population is calculated by solving a system of equations in partial derivatives, 1-d transport equations. The equations govern an evolution of neuronal states distributed in a phase space of the time elapsed since last spikes, t*. The system contains the Hodgkin-Huxley equations for the membrane voltage and gating variables, parameterized by t*, as well as the equation for the neuronal density in a t*-space (Chizhov, 2017).

The equations of the CBRD model were used to describe one excitatory and one inhibitory population. The excitatory population contains adaptive, regular spiking pyramidal cells with specific somatic and dendritic compartments with inhibitory synapses located at the soma and excitatory synapses at the dendrite. The inhibitory population contains fast-spiking, single-compartment neurons. The synaptic conductances are described by ordinary differential equations with the firing rate of a presynaptic population as an input signal. Through this approach, the AMPA- and NMDA-mediated conductances are controlled by the excitatory population firing rate, and the GABAa-conductance by the inhibitory population firing rate. The synapses were plastic. The weight that describes the short-term synaptic plasticity was approximated according to the Markram-Tsodyks model in its rate-based version (Loebel and Tsodyks, 2002).

The set of parameter values corresponded to those from a previous work (Chizhov et al., 2017) with the exception of the following (in the notation of the mentioned article): for the control case, the maximum synaptic conductances were *ḡ*_AMPA,E_ = *ḡ*_AMPA,I_ = 0.1 μS/cm^2^, *ḡ*_GABA,E_ = *ḡ*_GABA,I_ = 0.08 μS/cm^2^, *ḡ*_NMDA,E_ = *ḡ*_NMDA,I_ = 0.2 μS/cm^2^, the membrane area *S* = 0.7·10^−4^ cm^2^, the GABAaR reversal potential *V*_GABA_ = −70 mV, the extracellular magnesium concentration [Mg^2+^] = 1 mM; electrical contacts were missing. The conditions that precede epileptiform activity were the same as for the control case, except *ḡ*_NMDA,E_ = *ḡ*_NMDA,I_ = 0.16 μS/cm^2^, V_GABA_ = −55 mV [Mg^2+^] = 0.25. The epileptic conditions were modeled by setting the same parameter values as for the previous case, except *ḡ*_AMPA,E_ = *ḡ*_AMPA,I_ = 0.2 μS/cm^2^. For the sake of simplicity and to minimize the number of parameters, it was assumed that the values of synaptic conductances effectively take the synaptic resource to the moment of stimulation into account.

### Data Analysis and Statistics

The data analysis was performed using custom software written in Wolfram Mathematica 10 (Wolfram Research, Champaign, IL, USA). The statistical analysis and the graphical representation of the results were performed using Sigmaplot 12.5 (Systat Software Inc., San Jose, CA, USA). Dixon’s Q test (at a 90% confidence) was used to reject the outliners. The normality of sample data was evaluated by the Kolmogorov-Smirnov test. The equality of variance was assessed using the Levene median test. For data that had a normal distribution and passed an equal variance test, the statistical significance was assessed by a paired or an unpaired Student’s *t*-test, a one-way analysis of variance (ANOVA), or a repeated measures ANOVA where appropriate. The results were considered significant when *P* < 0.05. α was set equal to 0.05 for multiple comparison tests, which were performed only when ANOVA *P* value was significant. The Dunnett’s *post hoc* test was used for multiple comparisons vs. the control group. The Tukey *post hoc* test was used for multiple pairwise comparisons. The results are expressed as a mean ± standard error of the mean, *n* is a number of slices.

## Results

### Changes in the Relative Impact of AMPARs, NMDARs, and GABAaRs on Synaptic Responses After the Induction of Epileptiform Activity

Most studies have demonstrated that both excitatory and inhibitory neurons contribute to the generation of epileptic discharges (Huberfeld et al., 2011; Truccolo et al., 2011). The application of an extracellular stimulus to cortical slices generally results in the simultaneous activation of both types of neurons. Thus, first, seizure-induced changes in the contribution of excitatory and inhibitory synaptic conductances to evoked postsynaptic responses were investigated because they can reflect altered network properties during seizures.

**Figure 2:**
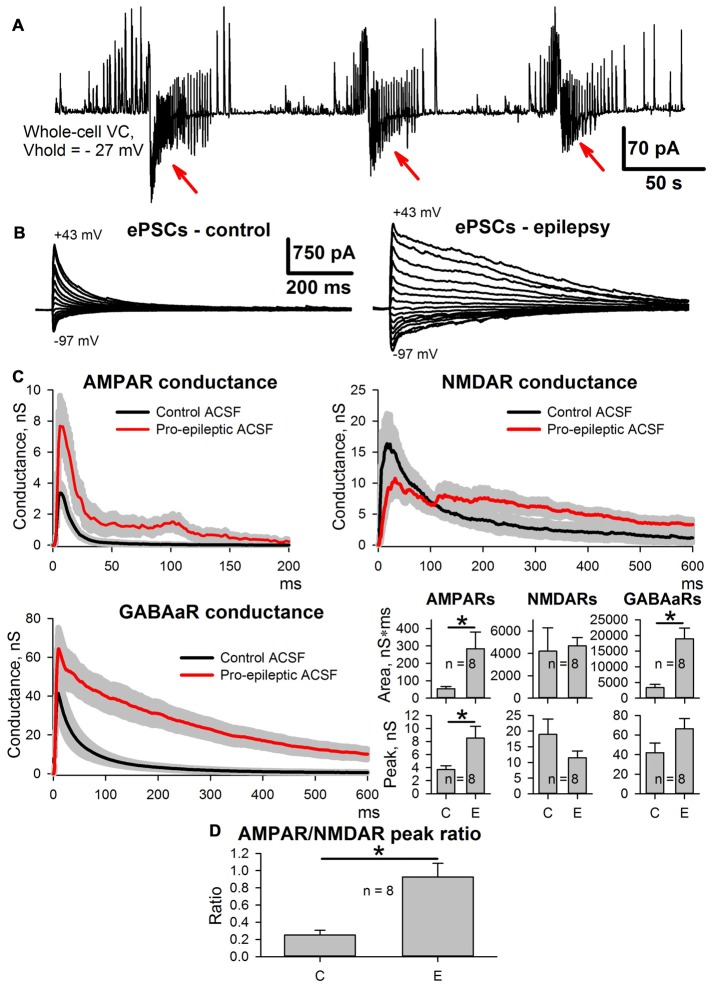
Changes in evoked postsynaptic current (ePSC) properties during epileptiform activity in entorhinal cortex (ERC). **(A)** Representative recordings of synaptic activity induced by a pro-epileptic solution in the ERC. Note the repeating patterns of current corresponding to seizure-like events (SLEs) discharges (marked with red arrows) in an entorhinal pyramidal neuron. **(B)** A representative example of two sets of ePSCs recorded from the same neuron at different holding voltages under control conditions (left) and during induced epileptiform activity in slices (right). **(C)** Estimates of AMPAR-, NMDAR-, and GABAaR-mediated components of the response to the extracellular stimulus under control conditions (black traces) and during epileptiform activity (red traces). Bar charts represent the areas under the plots and peak conductances (asterisks (*) indicate significant difference): epileptiform activity significantly increased the area and the peak of AMPAR-mediated conductance (paired Student’s *t*-test, *P* = 0.04 and 0.019 for peak and area, respectively) as well as the area of GABAaR-mediated conductance (paired Student’s *t*-test, *P* = 0.003). **(D)** The epileptiform activity increases the AMPAR/NMDAR peak conductance ratio (paired Student’s *t*-test, *P* = 0.006).

As reported previously (Amakhin et al., 2016), the perfusion of ERC slices with a pro-epileptic solution resulted in the appearance of several SLEs, which occurred every 1–5 min. A representative example of the voltage-clamp recording of SLEs is presented in Figure 2A. To investigate SLE-induced changes in the evoked responses, two sets of ePSCs were recorded at different voltages in ACSF (control conditions) and after the application of the pro-epileptic solution and 10–15 min of consequent epileptiform activity with at least three SLEs being detected (Figure 2B). The synaptic conductance estimation procedure was implemented for both sets of ePSCs (Figure 2C).

It was found that the monosynaptic peak amplitude and the area under the plot of AMPAR-mediated conductance evoked by extracellular stimulation changed after the induction of epileptiform activity (the average values of peak conductance and area under the plot increased by 130% and 427%, respectively). The peak value of GABAaR-mediated conductance did not change; however, its time course transformed, which resulted in a larger area under the conductance plot (the average value increased by 465%). No significant changes to the NMDAR-mediated peak conductance or the area under the conductance plot was detected, which resulted in an increased AMPA/NMDA peak conductance ratio (the average value increased 4.66-fold; Figure 2D); however, the time course of NMDAR conductance inactivation changed, leading to a significant increase in the conductance half-width (64 ± 12 ms in control conditions and 377 ± 67 ms during epileptiform activity, *n* = 8, *P* = 0.005, paired Student’s *t*-test).

### Potentiation of AMPAR-Mediated Synaptic Response Is Evoked by Epileptiform Activity, *per se*

**Figure 3:**
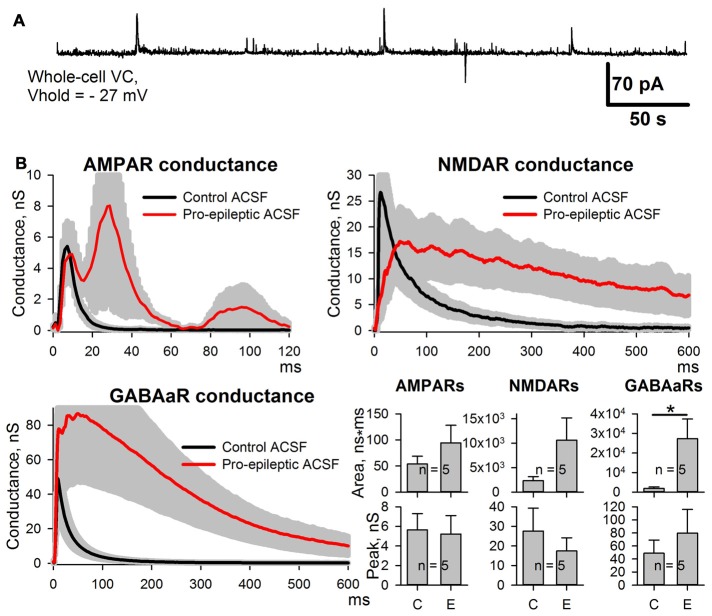
Changes of the AMPAR, NMDAR, and GABAaR components of the synaptic response of prefrontal cortex (PFC) neurons induced by the pro-epileptic solution. **(A)** Representative recording of synaptic activity induced by the pro-epileptic solution in the PFC. Under the same experimental conditions as in Figure 2A, no SLEs could be induced in PFC slices. **(B)** Estimates of AMPAR-, NMDAR-, and GABAaR-mediated components of the response to the extracellular stimulus under control conditions (black traces) and during perfusion with a pro-epileptic solution (red traces). Bar charts represent the areas under the plots and peak conductances: the perfusion of the ERC slices with a pro-epileptic solution resulted in a significant increase in the area of GABAaR-mediated conductance (paired Student’s *t*-test, *P* = 0.026, asterisk (*) indicates significant difference). No increase in AMPAR-mediated peak conductance was detected.

Next, we investigated which of the observed changes in ePSC properties are evoked by epileptiform activity *per se*, and which are the side effects of the pro-epileptic solution. The same experiments were performed with the PFC slices. In this brain region, the pro-epileptic solution failed to induce consistent epileptiform activity (Figure 3A). The same procedure of synaptic conductance estimation as in ERC was implemented (Figure 3B). No potentiation of the peak AMPAR-mediated conductance in PFC pyramidal neurons was found after a 25-min application of the pro-epileptic solution. The altered time course of GABAaR- and NMDAR-mediated conductances was reproduced in a PFC preparation without apparent epileptiform activity (the average area under the GABAaR-conductance plot increased by 1,340% and without a significant increase in the peak amplitude; NMDAR-conductance half-width increased from 56 ± 12 ms to 471 ± 135 ms (*n* = 5) in the control and pro-epileptic solutions, respectively; *P* = 0.04, paired Student’s *t-test)*. This indicates that only the potentiation of the AMPAR-mediated conductance in ERC is induced by epileptiform activity, while other detected changes emerge as a result of perfusion with the pro-epileptic solution.

### Potentiation of AMPAR-Mediated Synaptic Response Induced by Epileptiform Activity Was Transient

**Figure 4:**
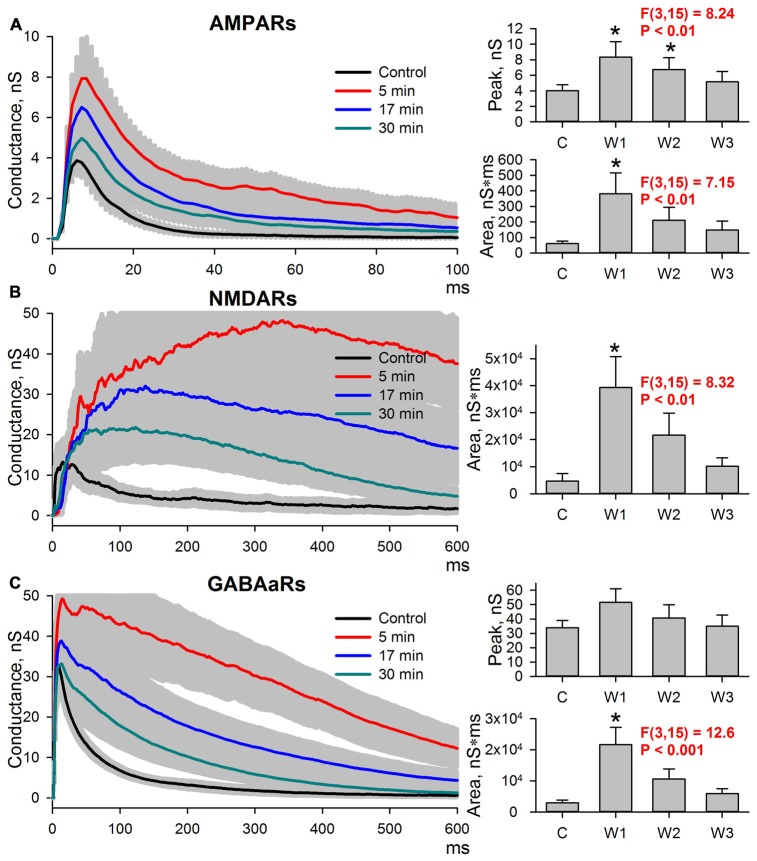
Changes in the AMPAR-, NMDAR-, and GABAaR-mediated components of the response persisted after the cessation of seizures. **(A–C)** Left panels: AMPAR- **(A)**, NMDAR- **(B)** and GABAaR- **(C)** components of the postsynaptic responses under control conditions and after the cessation of epileptiform activity. The estimates were performed at three time intervals from the cessation of seizures (5, 17, and 30 min). Right panels: bar charts that represent the areas under the plots and the peak conductances. Peak conductance for NMDARs was not estimated because no clearly distinguishable monosynaptic peak could be determined. AMPAR-mediated component remains augmented after seizures. “C,” “W1,” “W2,” and “W3” indicate control estimations and estimations at 5, 17 and 30 min after seizures, respectively. Asterisks indicate a significant difference from the control level (Dunnett’s *post hoc* test).

The length of time the potentiation of excitatory synaptic transmission persisted after the cessation of epileptiform activity was explored. Evoked responses in ACSF were recorded, and then the slices were perfused with a pro-epileptic solution to produce at least three SLEs. Next, the slices were washed with ACSF. In all slices, the epileptiform activity was abolished in less than 5 min after washout, and the sets of evoked responses were also recorded three times after washout: 5 min (W1), 17 min (W2), and 30 min (W3; Figure 4). All three postsynaptic conductances gradually returned to control values after washout. The peak AMPAR-mediated component of the response remained augmented at 5 and 17 min after the cessation of epileptiform activity compared to the control (the average values compared to control were increased by 107% and 68% for W1 and W2, respectively; repeated measures ANOVA, *P* = 0.002, followed by Dunnett’s *post hoc* test, Figure 4A).

### AMPAR-Mediated Synaptic Response Potentiation Is Dependent on NMDAR Activation

**Figure 5:**
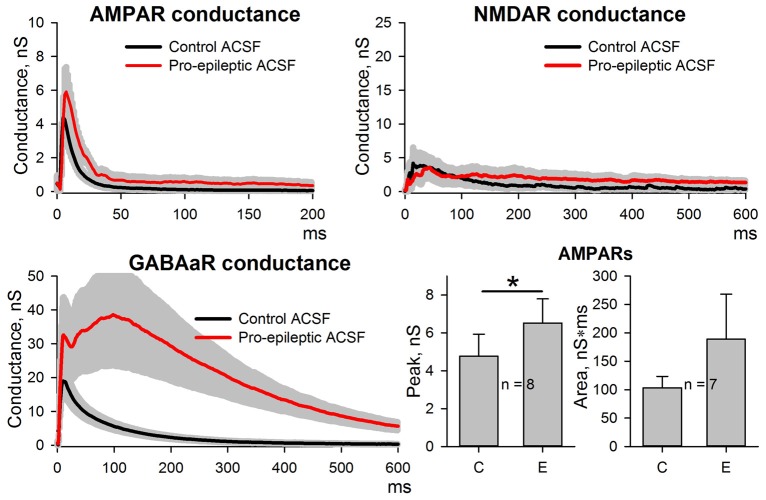
Changes in AMPAR, NMDAR, and GABAaR components of the synaptic response during epileptiform activity in ERC with a partial block of NMDA-receptors by intracellular MK-801. Top and bottom-left panels represent the estimates of AMPAR-, NMDAR-, and GABAaR-mediated components of the response to the extracellular stimulus under control conditions (black traces) and during epileptiform activity (red traces). Note the substantial block of NMDAR-mediated conductance by intracellular MK-801 (compare with Figure 2). Bar charts (bottom-right) represent the peak AMPAR-mediated conductance and the area under the plot: despite the partial block by intracellular MK-801, the response significantly increased (paired Student’s *t*-test, *P* = 0.03, asterisk (*) indicates significant difference); however, no changes in the area under the plot were detected (paired Student’s *t*-test, *P* = 0.27).

The potentiation of AMPAR-mediated conductance frequently depends on NMDAR activation (Chater and Goda, 2014). Therefore, whether the enhancement of AMPAR-mediated synaptic transmission by SLEs is dependent on the activation of NMDARs was investigated. In an attempt to avoid the alteration of epileptiform activity by adding the NMDAR antagonists to the perfusing solution, the recordings were performed with MK-801 (3 mM) added to the pipette solution. To increase the strength of the NMDAR block by MK-801, a low-frequency stimulation (0.05 Hz, 15 min) was performed before the conductance estimation protocol (Sun et al., 2018). Conductances were assessed in ACSF and during induced epileptiform activity. It was found that intracellular MK-801 decreased the amplitude of the NMDAR component about 5-fold but did not completely abolish it (Figure 5). Under these conditions, the increase in the AMPAR-mediated component peak conductance was still significant (the average value increased by 37%, Figure 5 bottom-left panel) but not as prominent as without an intracellular blockade of NMDARs (see Figure 2). No significant increase in the conductance plot area was observed (Figure 5 bottom left panel). The lack of a complete block of AMPAR-mediated component potentiation by intracellular MK-801 can be explained by the incomplete NMDAR blockade (Sun et al., 2018). As the results of this experiment were not totally conclusive, the NMDAR-dependence of AMPAR-mediated component potentiation was investigated using a modified model of *in vitro* epileptiform activity.

**Figure 6:**
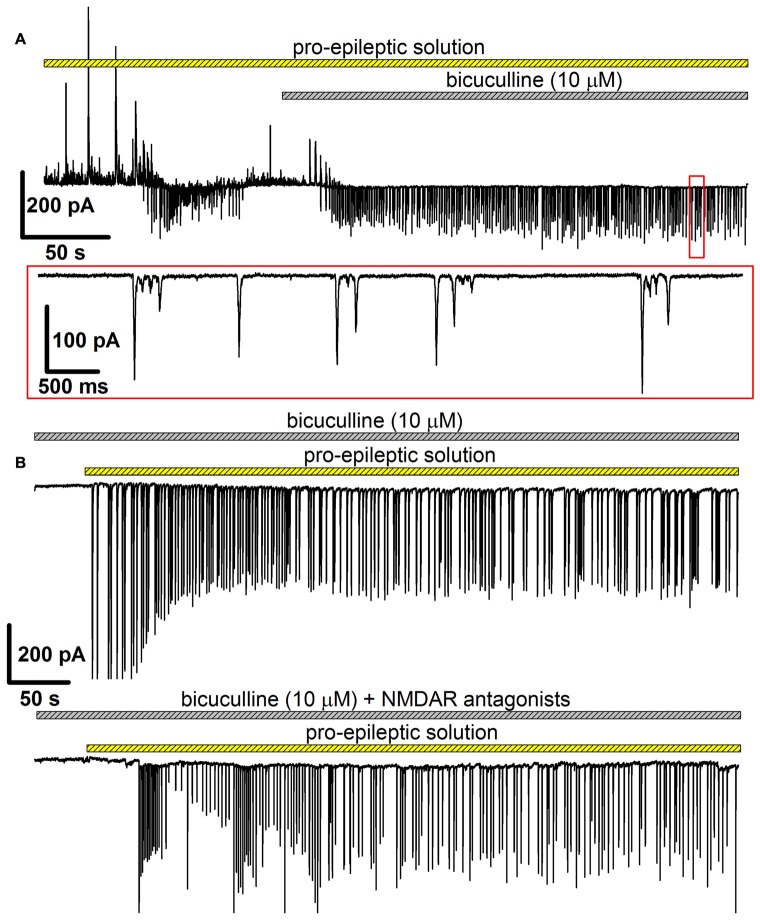
The alternative model of epileptiform activity, which was used to investigate the NMDAR-dependence of AMPAR potentiation. **(A)** A representative recording, which illustrates the difference between two models of epileptiform activity *in vitro*. The recording was initiated in the pro-epileptic solution (note the SLE at the first quarter of the upper trace). After the addition of bicuculline to the perfusing pro-epileptic solution, the pattern of synaptic activity changed: no SLEs could be observed, and regular glutamate-mediated events were present (the part of the recording marked with the red frame was extended on the lower trace). **(B)** Representative recordings of epileptiform activity in the ERC induced by the pro-epileptic solution with bicuculline. The epileptiform activity was roughly the same both without (upper trace) and with (lower trace) the blockage of NMDARs.

In this model, bicuculline (10 μM) was added to the pro-epileptic solution. The epileptiform activity manifested as high-amplitude regular-synchronized discharges; however, the typical SLEs were not reproduced (Figure 6). This model has two main advantages for investigating the effect of NMDARs on AMPAR potentiation. First, it allows for measuring AMPAR-mediated conductance directly by setting the holding voltage at −90 mV and assuming that at this voltage, the NMDAR conductance is negligible. Second, the synaptic activity pattern in this model was qualitatively identical with and without NMDAR antagonists present in the perfusing solution: in both cases, regular glutamatergic discharges were observed (Figures 6A,B), though the average frequency of the discharges was lower without the antagonists of NMDARs (the average frequency of discharges in the presence of bicuculline in a pro-epileptic solution was 0.47 ± 0.05 Hz (*n* = 13) without and 0.23 ± 0.03 Hz (*n* = 12) with NMDAR antagonists; *P* < 0.001, Student’s *t*-test).

**Figure 7:**
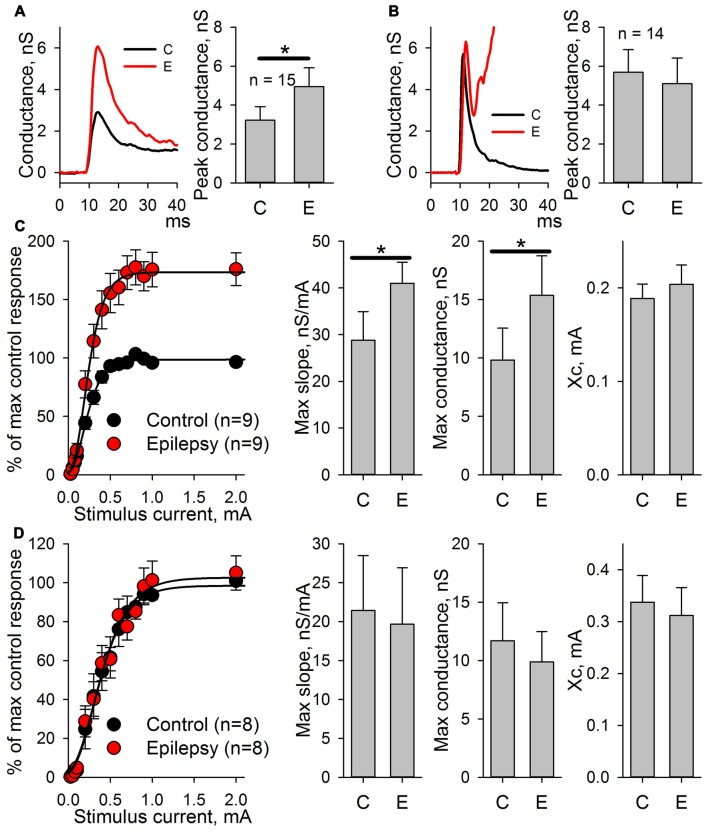
AMPAR-mediated evoked response amplitude increases after the induction of epileptiform activity in ERC in the presence of GABAR antagonists but not in the presence of GABAaR or NMDAR antagonists. **(A)** The AMPAR-mediated response of ERC neurons recorded in the presence of bicuculline in control ACSF (black line) and after the induction of epileptiform activity by a pro-epileptic solution with bicuculline (red line). The amplitude of the monosynaptic peak of the response increases (paired Student’s *t*-test, *P* < 0.001). **(B)** The AMPAR-mediated response of ERC neurons recorded in the presence of NMDAR antagonists and bicuculline in control ACSF (black line) and after the induction of epileptiform activity by a pro-epileptic solution with bicuculline and the NMDAR antagonists (red line). The amplitude of the monosynaptic peak of the response did not change (paired Student’s *t*-test, *P* = 0.33). **(C)** Left panel: the peak amplitude of AMPAR-mediated response vs. stimulus strength in control (black) and pro-epileptic conditions without the blockade of NMDARs (red). The data were normalized to the maximal response in control conditions and fitted with Gomperz function (Eq. 2). Right panel: the parameters of Eq. 2 under control conditions (C) and after the induction of epileptiform activity (E). A significant increase in the maximal slope of the curve and the maximal conductance *g*_max_ was detected (paired *t*-test, *P* = 0.002 and < 0.001, respectively; asterisks (*) indicate significant difference). **(D)** The same experimental protocol as in C was performed with the application of the antagonists of NMDARs. No significant changes in the parameters of Eq. 2 were detected (paired Student’s *t*-test).

First, whether the potentiation of AMPARs was preserved in this modified model was tested. eEPSCs were recorded in the presence of bicuculline both in ACSF and after the administration of the epileptogenic solution. Even minimal stimulation evoked polysynaptic responses in cortical neurons. Only the responses with a distinct monosynaptic peak observed about 4–7 ms after the stimulus were included in the analysis (Figures 7A,B). An increase in a peak AMPAR-mediated conductance after epileptiform activity was observed in the slices (the average value increased by 54%, Figure 7A, paired Student’s *t*-test, *P* < 0.001). The application of NMDAR antagonists prevented an increase in the peak value of AMPAR-mediated conductance (Figure 7B, paired Student’s *t*-test, *P* = 0.33).

The potentiation of AMPAR-mediated conductance should change the shape of I/O curves for eEPSCs to extracellular stimulation. Therefore, these shapes were compared based on AMPAR-mediated evoked responses in ACSF and after epileptic activity induced by applying the pro-epileptic solution in combination with bicuculline (Figure 7C) or bicuculline and NMDAR antagonists (Figure 7D). In both cases, the peak amplitudes of eEPSCs were plotted as a function of a stimulating current strength (Figures 7C,D, left panels). The resulting plots were fitted with the Gompertz function (Eq. 2), and changes in its parameters were evaluated. Epileptiform activity increased the value of *g*_max_ (the average value increase by 42%) and the maximal slope of the curve (the average value increased by 56%) but not *X*_c_ (Figure 7C, right panel). The addition of NMDAR antagonists to the pro-epileptic solution completely preserved the shape of the I/O curve (Figure 7D, right panel). The results of these experiments indicated that the potentiation of the AMPAR-mediated response depends on the activation of NMDARs.

### Epileptiform Activity Causes the Increase of AMPAR-Mediated Miniature Currents’ Amplitudes

**Figure 8:**
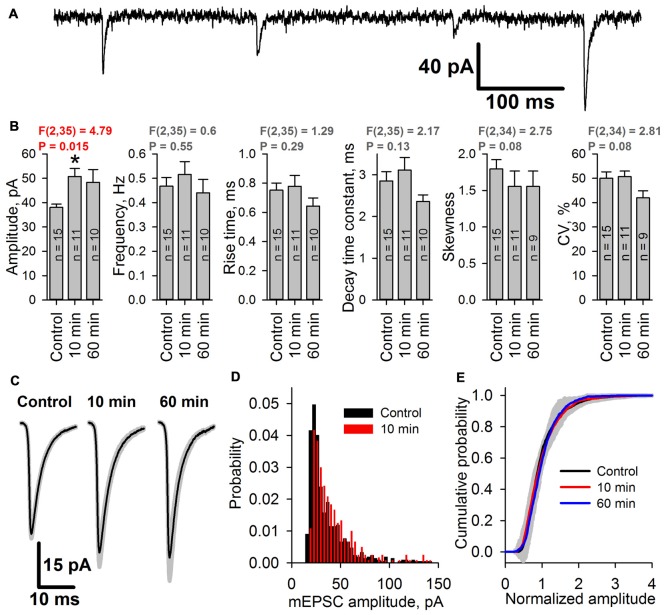
The amplitude of miniature AMPAR-mediated current increases after SLEs. **(A)** A representative recording of miniature excitatory postsynaptic currents (mEPSCs; *V*hold = −80 mV). **(B)** The average amplitude but not the average frequency of mEPSCs increases after epileptiform activity [one-way analysis of variance (ANOVA)]. The asterisk indicates a significant difference from the control group, Dunnet’s *post hoc* test. **(C)** Average mEPSCs recorded under control conditions, 10 min, and 60 min after seizures. No changes in the mEPSC rise or decay kinetics were detected. **(D)** Representative examples of mEPSC amplitude distributions in pyramidal neurons from control (black) and 10 min group (red). **(E)** Average cumulative distributions of the normalized mEPSC amplitudes. A gray area indicates the standard deviation of the mean cumulative probability.

To assess whether the increased peak AMPAR conductance is due to the elevated glutamate release probability or changes in the postsynaptic membrane, the properties of mEPSCs in pyramidal neurons were analyzed before and after an hour-long preincubation of the slices in the pro-epileptic solution at 30°C. The properties of the mEPSCs were analyzed 10 and 60 min after transferring the slices from the pro-epileptic solution to ACSF (Figure 8). No changes in the frequency of mEPSCs compared to the control were observed (Figure 8B), meaning that the glutamate release probability from the presynaptic terminals remained unaltered following the washout of pro-epileptic solution; however, preincubation in the pro-epileptic solution increased the amplitude of the mEPSCs for at least 10 min (at 10 min, the average value was increased by 33% relative to the control; Figure 8B), but no significant changes in mEPSC rise time or decay time constant were detected (Figures 8B,C). These results suggest that mostly postsynaptic mechanisms underlie the elevation of AMPAR conductance after a period of epileptiform activity in ERC slices.

As the epileptiform activity is unlikely to equally affect all of the synaptic inputs on the neurons in brain slices, we hypothesized that the AMPAR potentiation might occur only in a certain subset of synapses. That would change the shape of the distribution of mEPSC amplitudes, leading to the increased prominence of its right tail, increased coefficient of variation, and the changed coefficient of skewness. However, we found that the shapes of mEPSC amplitude distributions before and after SLEs look the same (Figure 8D); and both coefficients have not changed (Figure 8B). To further investigate the possible differences in the mEPSC populations, we compared the average cumulative distributions of normalized mEPSCs for each group (Figure 8E). To do this, we normalized the mEPSC amplitudes by their mean for each cell, plotted the cumulative distributions of the resulting data, and then averaged those distributions for each group. The resulting distributions for 10 min and 60 min groups were identical to control one, which was confirmed by the Kolmogorov-Smirnov test. These results suggest that the mEPSC amplitude potentiation occurs relatively uniformly across all active synapses in the neuron.

### Epileptiform Activity Promotes the Incorporation of Calcium-Permeable AMPA-Receptors in the Synapses of ERC Pyramidal Neurons

Some forms of synaptic plasticity are accompanied by transient incorporation of calcium-permeable AMPA-receptors (CP-AMPARs) in excitatory synapses (Plant et al., 2006; Rajasekaran et al., 2012). The authors’ previous study demonstrated that in the pilocarpine model of epilepsy, the incorporation of CP-AMPARs in the excitatory synapses of cortical pyramidal neurons occurs 3 days after SE (Malkin et al., 2016). Here, whether the observed increase in AMPAR conductance during *in vitro* epileptiform activity is accompanied by the increase of the relative impact of CP-AMPARs in postsynaptic responses was investigated.

**Figure 9:**
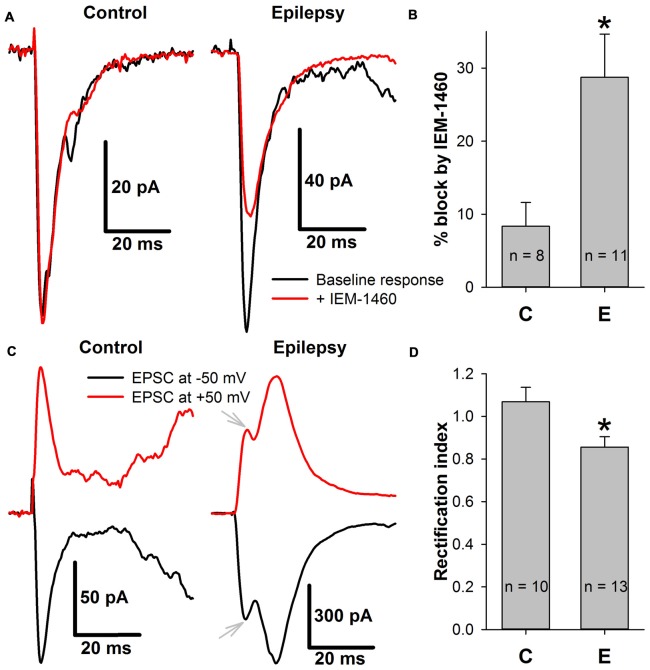
The incorporation of calcium-permeable AMPA-receptors (CP-AMPARs) in the postsynaptic membrane of ERC pyramidal neurons. **(A)** Representative examples that illustrate the effect of IEM-1460 on AMPAR-mediated evoked responses under control conditions (left) and during epileptiform activity (right). Note the increased effect of the antagonist under pro-epileptic conditions. **(B)** IEM-1460 exerts a significantly stronger effect on the AMPAR-mediated response after a period of epileptiform activity (Student’s *t*-test, *P* = 0.014, asterisk (*) indicates significant difference). **(C)** Representative examples of AMPAR-mediated eEPSCs recorded at −50 and +50 mV under control conditions (left) and during epileptiform activity (right). Note the mismatch between the positive and negative-going response under pro-epileptic conditions. Gray arrows mark monosynaptic peaks which were used for RI calculation. **(D)** The rectification index of AMPAR-mediated eEPSC significantly decreased after a period of epileptiform activity (Student’s *t*-test, *P* = 0.017, asterisk (*) indicates significant difference).

First, whether the efficiency of the blockade of the AMPAR-mediated eEPSCs by a selective blocker of CP-AMPARs, IEM-1460 (Samoilova et al., 1999), changed in ERC pyramidal neurons after a period of epileptiform activity was explored (Figures 9A,B). It was found that the blocking effect of IEM-1460 (100 μM) increased 3.44-fold relative to the control conditions (Figure 9B).

CP-AMPARs display inwardly rectifying I-V relationships (Bochet et al., 1994; Zaitsev et al., 2011). Thus, the AMPAR-mediated eEPSCs were recorded at −50 mV and +50 mV, and the rectification index (Figures 9C,D) was calculated as described in the Methods section. A significant decrease in the rectification index after epileptiform activity (RI decreased by 20% relative to the control conditions, Figure 9D). Taken together, these results indicate that SLEs lead to the inclusion of CP-AMPARs in the postsynaptic membrane of pyramidal cells.

### AMPAR Potentiation Increases the Duration of the Firing Response of Pyramidal Neurons on Extracellular Stimulation

**Figure 10:**
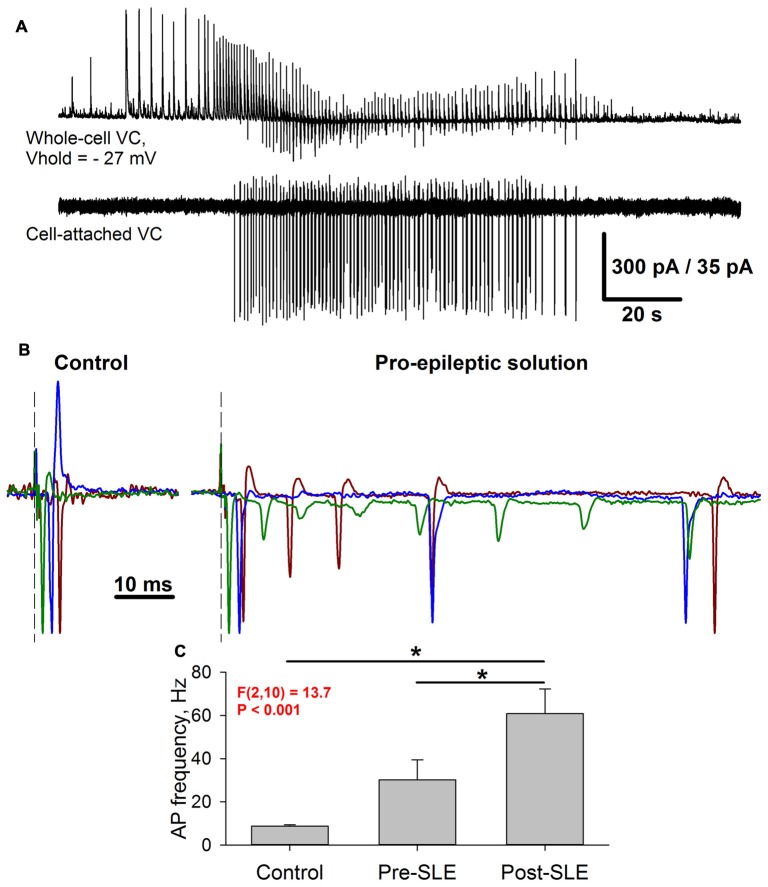
Seizure-induced changes of pyramidal cell firing activity. **(A)** Simultaneous recording of two neighboring pyramidal neurons during SLEs. Upper trace: a representative recording of synaptic activity in the voltage-clamp mode at −27 mV. Lower trace: a corresponding firing activity of the neighboring neuron recorded in the cell-attached voltage-clamp mode. **(B)** Three superimposed representative recordings in a cell-attached voltage-clamp configuration under control conditions (left) and the pro-epileptic solution (right). The current amplitude was normalized to the amplitude of the first recorded action potential. Identical colors correspond to the same recorded cell. Note the marked increase in the action potential (AP) number in the pro-epileptic solution. **(C)** Average action potential frequency over a 100-ms interval from the stimulus under control conditions, in a pro-epileptic solution before the first SLE (Pre-SLE), and after a period of robust epileptiform activity (Post-SLE). Note the increase in AP frequency following a period of epileptiform activity (one-way repeated measures ANOVA; asterisks indicate a significant difference between groups, Tukey test).

It was demonstrated that GABAaR-mediated conductance prevails during burst discharges, which constitute the SLEs (Amakhin et al., 2016). Evoked responses share this property with spontaneous epileptiform discharges due to the polysynaptic activation of GABAergic neurons. Next, the way the strengthening of AMPAR-mediated synaptic transmission modifies the firing activity of pyramidal neurons in response to extracellular stimulation was investigated. To do so, the evoked responses in the cell-attached voltage-clamp mode under control and pro-epileptic conditions were examined (Figure 10). When being recorded in this mode, the SLEs manifested themselves as a salvo of action potentials, such as those illustrated by Figure 10A.

Under control conditions, the stimulus generally induced a single action potential, which roughly coincides with the peak of AMPAR-mediated evoked conductance (Figure 10B, left panel); however, under pro-epileptic conditions, the extracellular stimulation of the same magnitude resulted in a burst of 2–10 action potentials (Figure 10B, right panel) despite the domination of GABAaR-mediated conductance during the response. To investigate the effect of AMPAR-potentiation on the firing response of pyramidal cells, the average action potential frequency over a 100-ms period was calculated from the stimulus under control conditions after 3–5 min of perfusion with the pro-epileptic solution (but before the first SLE) and after the induction of robust epileptiform activity (Figure 10C). It was found that after epileptiform activity, the cells were able to generate a higher firing frequency in response to stimulation than before it (a 2-fold increase of the average firing rate after a period of epileptiform activity was observed). These results indicate that the observed augmentation of excitatory synaptic transmission might lead to a higher action potential probability in pyramidal neurons despite the strong concurrent activation of GABAaRs.

### Simulation of the Evoked Responses

During the experiments, it was difficult to separate the roles of AMPAR-potentiation and factors contributing to complex network activity. The results (Figures 10B,C) indicate that the augmentation of the AMPAR-mediated conductance might be a factor that promotes the increase in the pyramidal neuron firing rate in response to extracellular stimulation; however, it is unclear whether it is the only contributing factor, and the minimal set of conditions that underlies the observed ePSC transformation under pro-epileptic conditions is unknown.

To clarify this issue, a mathematical model of interacting excitatory and inhibitory populations was used. The model was taken from the authors’ previous modeling study of interictal discharges (Chizhov et al., 2017). In the previous study, this model allowed for reproducing the two main types of spontaneous epileptiform discharges in ERC, so it was implemented to simulate the evoked responses.

**Figure 11:**
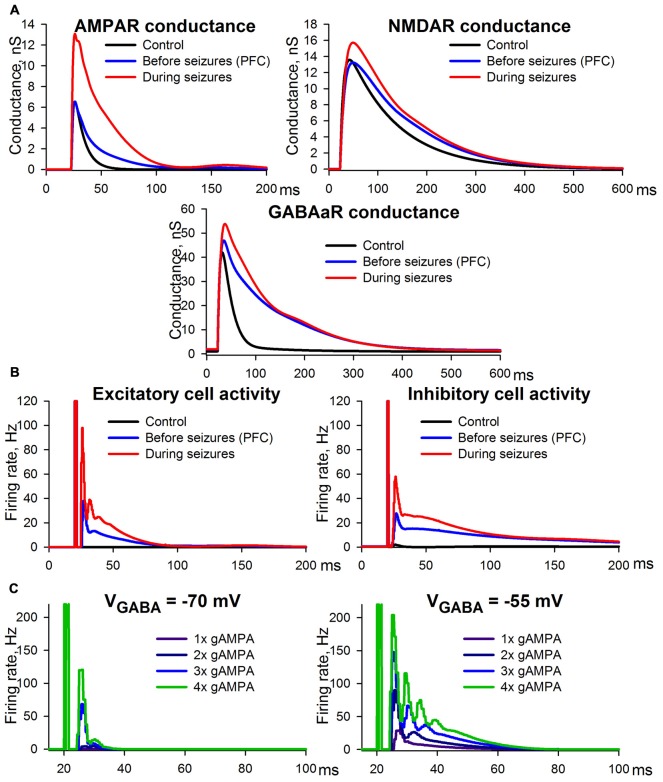
Simulation of ePSCs using the CRBD approach. **(A)** Synaptic conductance components. **(B)** Firing rates of excitatory and inhibitory populations. The traces obtained under control conditions and epileptic conditions are to be compared with those in Figure 2C, while those obtained under the conditions preceding SLEs are to be compared with Figure 3B. **(C)** The effects of AMPAR enhancements under the conditions of a normal (left panel) and a decreased (right panel) transmembrane gradient of chloride ions.

The control conditions were modeled using the following settings: (i) GABAaR-current reversal potential was −70 mV; (ii) extracellular magnesium concentration was 1 mM as in ACSF; and (iii) AMPAR-, NMDAR-, and GABAaR-mediated maximum conductances were selected so that the resulting simulations of synaptic conductances (Figure 11A) were close to the experimental estimations (Figure 2C).

The experimental conditions that preceded the epileptiform activity were assumed to be identical to the situation in which PFC slices were perfused with a pro-epileptic solution (Figure 3B). The following settings were selected to reproduce this experimental situation: (i) GABAaR reversal potential was enhanced up to −55 mV due to the increased potassium concentration in the pro-epileptic solution, which leads to the downregulation of KCC2 exchangers (Buchin et al., 2016; Doyon et al., 2016; Di Cristo et al., 2018); (ii) extracellular magnesium concentration was 0.25 mM, as in the pro-epileptic solution; and (iii) AMPAR- and GABAaR-mediated maximum conductances were identical to those under the control conditions, while in order to reproduce our experimental results NMDAR-mediated maximum conductance was decreased by 20% compared to the control conditions. This decrease was introduced under the assumption that the fraction of functionally available NMDARs is reduced due to an increased ambient level of glutamate (Le Meur et al., 2007; Featherstone and Shippy, 2008), which might be the case during perfusion with the pro-epileptic solution (Hozumi et al., 2011; Kanamori and Ross, 2011; Medina-Ceja et al., 2015). Using these settings, the model reproduced the prolonged kinetics of the AMPAR-, NMDAR-, and GABAaR-mediated components of the response due to recurrent polysynaptic excitation. As in the experimental estimations, the simulated peak AMPAR- and NMDAR-conductances during the evoked response did not change.

Epileptic conditions were modeled using the same settings as the conditions that preceded epileptiform activity, except that the AMPAR-mediated maximum conductance was twice as large as that of the control conditions due to the potentiation of postsynaptic AMPARs. This simulation yielded results consistent with the experimental observations. The peak AMPA conductance under epileptic conditions was more than twice as large as that of the control (compare the top panel in Figure 11A to the top-left panel in Figure 2C). This effect is a consequence of the doubled maximum conductance and the recurrent polysynaptic excitation. The recurrent excitation also manifested itself as a dramatically changed firing rate (the top panel in Figure 11B). The peak GABA conductance increased moderately, but the kinetics was significantly prolonged (compare the bottom panel in Figure 11A to the bottom-left panel in Figure 2C), which is in accordance with the firing rate (the bottom panel in Figure 11B). The peak NMDA conductance did not change significantly, but the kinetics were prolonged (compare the middle panel in Figure 11A to the top-right panel in Figure 2C), which reflects the opposing effects of the recurrent excitation and the hypothesized downregulation. In sum, the simulation revealed that the mechanism of ePSC transformations under epileptic conditions consists in the onset of the recurrent polysynaptic excitation that follows super-threshold monosynaptic excitation mediated by AMPARs. Thus, the simulation demonstrated that only two non-trivial factors may underlie the transformation of ePSC during epileptiform activity: the AMPAR potentiation and a slight down-regulation of NMDARs.

To highlight the potential role of seizure-induced AMPAR-potentiation, its effects on the firing rate of pyramidal cells were examined under control conditions and under conditions with enhanced GABAaR-current reversal potential, which can result from altered ionic homeostasis during SE (Raimondo et al., 2015; Figure 11C). The simulations demonstrated that AMPAR enhancement induces a much stronger increase in the firing rate under the conditions of an altered transmembrane chloride gradient than under normal conditions. Under normal conditions even with strong, 4-fold potentiation of AMPARs, the pyramidal neurons were capable of firing only within a relatively short time interval after the stimulus. In the case of a decreased chloride gradient, the pyramidal neurons were able to generate a higher firing rate in response to stimulation, and the time interval of firing was substantially prolonged with each incremental increase in AMPAR conductance. These results indicate that the potentiation of AMPARs may be a key mechanism in increased seizure susceptibility during SE.

## Discussion

### AMPAR Plasticity as a Consequence of Seizures

AMPAR-mediated synaptic transmission properties are highly variable and can be regulated in an activity-dependent manner at different levels: the number of AMPARs in synapses, their subunit composition, the phosphorylation state, and interactions with intracellular molecules (Chater and Goda, 2014). Our results demonstrate that even several minutes of epileptiform activity (three SLEs) lead to the transient potentiation of postsynaptic AMPARs in ERC pyramidal neurons, which is accompanied by an increase in the relative impact of CP-AMPARs on the postsynaptic response. The NMDAR-dependence of the AMPAR potentiation indicates that it shares a common mechanism with conventional LTP. This observed similarity is in agreement with previous reports on the pyramidal neurons of the hippocampus in which different models of epileptic seizures were implemented (Abegg et al., 2004; Debanne et al., 2006; Joshi et al., 2017).

**Figure 12:**
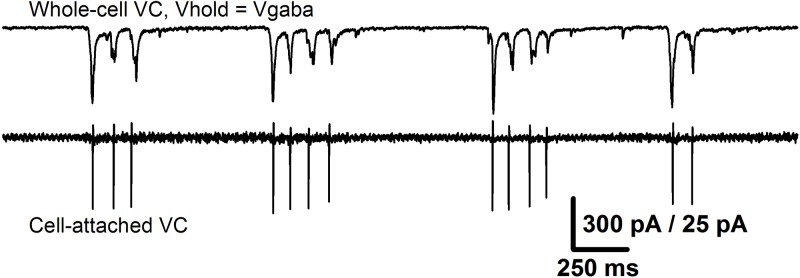
Epileptiform activity in the ERC resembles a burst stimulation protocol. The recordings from the same pair of neurons as in Figure 10A that represent the short bursts of synchronized activity that constitute SLE. Upper trace: the recording of glutamatergic synaptic input (holding potential was set equal to the reversal potential of GABAaR-mediated currents). Lower trace: the corresponding firing activity of the neighboring neuron. Note that the number of action potentials in all short bursts is equal to the number of peaks of glutamate-mediated currents.

Conventional LTP is typically induced by a theta-burst stimulation, which results in the AMPAR-mediated depolarization of the postsynaptic membrane and the consequent activation of NMDARs. The latter mediates the influx of Ca2^+^– ions, which initiates a series of phosphorylation events leading to an increase in the excitatory postsynaptic response strength (Chater and Goda, 2014). It is well-established that a burst stimulation with a broad frequency range can cause the potentiation of excitatory synapses in the hippocampus (Grover et al., 2009). The perfusion of ERC slices with a pro-epileptic solution generally leads to SLEs, which in this preparation consisted of 20–80 short bursts of activity with an average frequency of about 0.5–3 Hz. Simultaneous recordings of two neighboring ERC neurons in whole-cell and cell-attached configurations (Figure 12) revealed that during each short burst in most pyramidal neurons, two to five action potentials were generated synchronously with glutamatergic synaptic input. Taken together, these observations indicate that epileptiform activity results in the synchronous firing of a large number of glutamatergic neurons, which somewhat resemble the burst stimulation protocol for LTP induction (though it has a smaller burst frequency than the generally utilized theta-burst stimulation; Grover et al., 2009; Larson and Munkácsy, 2015). It is highly likely that such a pattern of activity leads to the potentiation of AMPARs in ERC neurons through the same well-established mechanism as the hippocampal LTP.

### Incorporation of CP-AMPARs in ERC Pyramidal Neurons

The obtained results indicate that even 10 min of epileptiform activity in ERC slices is sufficient to induce the incorporation of CP-AMPARs in pyramidal neurons. Estimations of the effect of IEM-1460 on the evoked current revealed that newly emerged CP-AMPAR-mediated conductance constitutes a substantial part of the overall augmentation of the AMPAR response.

The link between either seizures or epileptiform activity and changes in the expression of GluA2 at the hippocampal synapses have been demonstrated in a number of studies (Grooms et al., 2000; Huang et al., 2002; Borbély et al., 2009; Rajasekaran et al., 2012; Malkin et al., 2016). The latter work by Rajasekaran et al. (2012) focuses special attention on AMPAR plasticity in a pilocarpine model of SE and confirms the expression of GluA2-lacking AMPARs both by electrophysiological and biochemical methods; however, the precise role of CP-AMPARs in sustaining epileptic seizures or epileptogenesis is still poorly understood. To the authors’ knowledge, there is no data regarding the changes in GluA2 expression or an enhancement in AMPAR-mediated currents at the synapses of entorhinal neurons during induced epileptiform activity *in vitro*. The implementation of an *in vitro* model of epileptic seizures allowed for demonstrating the expression of CP-AMPARs within minutes after the last SLE, indicating that a highly rapid mechanism of their incorporation in synapses is activated during epileptiform activity. Such rapid incorporation of a substantial amount of CP-AMPARs may play an essential role in seizure-induced plastic changes in the parahippocampal region circuitry because it has been demonstrated that some forms of synaptic plasticity are mediated entirely by CP-AMPARs (Wiltgen et al., 2010).

### AMPAR Potentiation Is One of the Key Determinants of Increased Network Excitability During Seizures

The authors’ studies (Amakhin et al., 2017) and previous studies conducted by other groups (Ben-Ari and Gho, 1988; Bains et al., 1999; Gutiérrez, 2000; Lopantsev et al., 2009) showed significant changes of synaptic transmission properties following epileptic discharges, which manifest themselves in altered characteristics of the evoked postsynaptic responses. Simulations using the CBRD approach reveal a minimal set of modified parameters required to alter the synaptic transmission properties so that the network can reproduce both spontaneous epileptiform discharges (Chizhov et al., 2017) and the most prominent features of mixed GABAergic and glutamatergic evoked postsynaptic response. A significant increase in AMPAR conductance and a moderate decrease in NMDAR conductance were required to reproduce the evoked response alterations in addition to the direct consequences of the pro-epileptic solution application: the decrease in the magnesium blockage of NMDARs and the depolarizing effect of GABAaRs.

A positive shift in *V*_GABA_, introduced in the simulations, takes into account the dynamic changes in this parameter during a seizure (Fujiwara-Tsukamoto et al., 2003). In adult animals, the dynamics of this parameter is determined primarily by the balance between intracellular chloride accumulation via GABAaRs (Staley et al., 1995) and subsequent extrusion by cation-dependent chloride transporters such as KCC2 (Rivera et al., 1999). Whereas in low extracellular potassium solution, since tens of seconds after an SLE the GABAergic components switch their polarity from depolarizing to hyperpolarizing (Fujiwara-Tsukamoto et al., 2003), a rise in extracellular potassium in our experimental conditions slows down the process of chloride extrusion by means of inhibition or even reversal of the chloride extruder KCC2 (Thompson and Gähwiler, 1989; Jensen et al., 1993; Payne et al., 1996; DeFazio et al., 2000; Kakazu et al., 2000). Though the shift in *V*_GABA_ due to elevation of extracellular potassium in the steady state of a silent network is moderate (Isaev et al., 2007), it is high during SLEs (Fujiwara-Tsukamoto et al., 2003) due to intracellular chloride accumulation via GABAaRs, and remains to be significantly enhanced in the intervals between SLEs (Chizhov et al., 2017) and some minutes after the discharges. In our model, we assumed V_GABA_ to be shifted from −70 mV in control to −55 mV in epileptic conditions, thus approximating an average V_GABA_ level in the intervals between SLEs.

It is well established that during prolonged SE both patients and experimental animals often lose their sensitivity to positive modulators of GABAaRs such as benzodiazepines (Walton and Treiman, 1988; Treiman et al., 1998; Jones et al., 2002). These findings indicate that during seizures the pharmacological enhancement of GABAaR function either does not result in the strengthening of inhibition of neuronal firing due to altered chloride gradient or is insufficient due to the potentiation of AMPARs. Recent reports demonstrate that cerebrospinal levels of neurosteroids such as allopregnanolone which are also known to enhance GABAaR currents, are reduced in patients affected by SE (Meletti et al., 2017, 2018), indicating a potential downregulation of GABAaRs which might even further decrease the effectiveness of inhibition in patients.

No changes in GABAaR conductance were required in the model, which is consistent with some of the previous experimental studies (Esclapez et al., 1997; El-Hassar et al., 2007). However, some studies demonstrate the depression of GABAaR-mediated transmission after short-lasting epileptiform activity in the hippocampus (Le Beau and Alger, 1998; Evans et al., 2006; Lopantsev et al., 2009), indicating that the utilized experimental model of seizures might be of importance. The introduction of decreased NMDAR conductance in the model might be explained by the decreased fraction of available NMDARs due to their partial desensitization or tonic activation due to high levels of extracellular glutamate (Featherstone and Shippy, 2008). The latter could be the result of increased glutamate release during perfusion with a pro-epileptic solution because a significant increase in NMDAR-mediated conductance was observed during the washout (Figure 4). Due to their high sensitivity to glutamate, NMDARs should be more affected by its ambient concentration than AMPARs. Though there are contradicting reports concerning the levels of extracellular glutamate in brain slices and its ability to activate synaptic receptors under normal conditions (Herman and Jahr, 2007; Le Meur et al., 2007; Herman et al., 2011), we hypothesize that the implementation of a pro-epileptic solution can result in higher ambient levels of this neurotransmitter, leading to its stronger effects on NMDARs. Overall, this study reveals that AMPAR potentiation is a factor that shifts the balance of excitation and inhibition in an epileptic state and thus must be accounted for in experimental and modeling considerations.

The precise consequences of seizure-induced AMPAR potentiation are still not fully characterized, though it has been hypothesized that it helps sustain prolonged seizures (Joshi and Kapur, 2018). The simulation provides evidence in favor of this hypothesis by demonstrating that in combination with an altered chloride gradient (which is highly likely to be the case during SEs (Raimondo et al., 2015), such potentiation can substantially promote the spread of excitation over the neural network.

## Data Availability

The datasets generated for this study are available on request to the corresponding author.

## Author Contributions

DA, AC and AZ designed the study, interpreted data for the work and wrote the manuscript. DA, ES, JE and SM performed experiments and analyzed data. AC developed the mathematical model and performed the simulations. DA, ES, JE, SM, AC and AZ approved the final version.

## Conflict of Interest Statement

The authors declare that the research was conducted in the absence of any commercial or financial relationships that could be construed as a potential conflict of interest.

## Footnotes

^1^http://scipy.org
